# Cutting Edge: Circulating Plasmablasts Induce the Differentiation of Human T Follicular Helper Cells via IL-6 Production

**DOI:** 10.4049/jimmunol.1401190

**Published:** 2015-02-13

**Authors:** Konstantia-Maria Chavele, Eve Merry, Michael R. Ehrenstein

**Affiliations:** Centre for Rheumatology, Division of Medicine, University College London, London WC1E 6JF, United Kingdom

## Abstract

B cells require CD4^+^ T follicular helper (Tfh) cells to progress through the germinal center and provide protective Ab responses. In this article, we reveal a reciprocal interaction whereby circulating human plasmablasts are potent inducers of the Tfh cell–differentiation program, including the expression of their key transcription factor Bcl-6. The markedly increased propensity of plasmablasts, compared with naive B cells, to induce Tfh cell differentiation was due to their increased production of IL-6. Specific targeting of IL-6 using tocilizumab therapy in patients with rheumatoid arthritis led to a significant reduction in circulating Tfh cell numbers and IL-21 production, which was correlated with reduced plasmablast formation. Our data uncover a positive-feedback loop between circulating plasmablasts and Tfh cells that could sustain autoimmunity and spread Ab-driven inflammation to unaffected sites; this represents an important therapeutic target, as well as reveals a novel mechanism of action for tocilizumab.

## Introduction

CD4^+^ T follicular helper (Tfh) cells are a specialized Th subset that provides signals to B cells and guides their development through the germinal center (GC) (1). This Tfh cell–B cell interaction lies at the heart of the GC and is key for efficient immune responses; however, it also can promote autoimmune disease. Tfh cells express the transcriptional repressor Bcl-6; produce IL-21, which is essential for robust high-affinity humoral responses; and express ICOS and CXCR5, the latter directing this cell lineage to the GC. B cells with the highest affinity for Ag present cognate peptides to Tfh cells and, in turn, receive critical signals allowing their survival and differentiation into memory B cells and plasma cells. The differentiation of Tfh cells is reliant upon multiple signals, including cytokines, such as IL-6 and IL-21 (2, 3). Murine B cells can reciprocally modulate Tfh cell dynamics and promote their formation, such as by the provision of IL-6. In contrast, little is known about the role of B cells in human Tfh cell differentiation. In this article, we investigate the role of B cells in the induction of human Tfh cells.

## Materials and Methods

### Healthy individuals and patients

Blood was obtained from healthy individual volunteers and patients with rheumatoid arthritis (RA) before and 6 mo after treatment with tocilizumab. The mean disease activity score (DAS28) in the paired samples before and after tocilizumab treatment was 7.2 and 3.5, respectively. These patients were not taking any other disease-modifying antirheumatic drugs and were on <7.5 mg prednisolone/d. The University College London Hospital ethics committee approved the study. The *n* values in the figure legends refer to independent donors.

### Human cell isolation

B cells were depleted from PBMCs by positive selection with magnetic beads (Miltenyi Biotec). Responder and naive T cells were sorted as CD4^+^CD25^−^CD127^+^ and CD4^+^CD45RA^+^CD27^+^, respectively. B cell subpopulations, including plasmablasts and naive and memory B cells, were sorted as CD19^+^CD38^+^CD27^+^, CD19^+^IgD^+^CD38^−/int^CD27^−^, and CD19^+^CD38^−^CD27^+^ respectively.

### Cell culture

PBMCs were stimulated with 2 μg/ml soluble anti-CD3 (HIT3a) and anti-CD28 (CD28.2) (eBioscience). A total of 50,000 cells each was used for cocultures of T and B cells. For Tfh functional assays, CD4^+^ T cells were resorted after 4 d of culture, with or without plasmablasts, and cultured with autologous freshly sorted naive B cells in the presence of 2 μg/ml endotoxin-reduced Staphylococcal enterotoxin B (Sigma-Aldrich). Naive T cells were cultured with 10 ng/ml of IL-21 or IL-6 or a combination of both (PeproTech).

### Abs

The following Abs were used: CD4–Alexa Fluor 700, CXCR5-Biotin, ICOS-PECy7, Bcl-6–Alexa Fluor 647, CD27–allophycocyanin–H7, CD45RA-PE, CD19-allophycocyanin or V450, IgD-FITC, IL-21–Alexa Fluor 647, IFN-γ–PECy7, IL-10–PE, Streptavidin–PE–Texas Red (BD Biosciences), CD38–PerCP–eFluor 710, and IL-6–FITC (BioLegend). Neutralizing Abs specific for human IL-6 and IL-21R and isotype controls were from R&D Systems.

### Flow cytometry

For analysis of intracellular cytokines, cells were stimulated for 4 h with 50 ng/ml PMA, 250 ng/ml ionomycin (Sigma-Aldrich), and GolgiPlug (BD Biosciences). Data were acquired on an LSR II (Becton Dickinson) and analyzed with FlowJo software (TreeStar).

### ELISA

IL-6 (eBioscience) and Ig (IgM and IgG) production (Sigma-Aldrich) was measured in supernatants by ELISA, according to the manufacturers’ instructions.

### Statistical analysis

Data were analyzed for significance by the paired *t* test using Prism (GraphPad, La Jolla, CA). The Mann–Whitney *U* test was used to compare healthy individuals and patients with RA. Correlation coefficients and their significance were analyzed by the Pearson correlation.

## Results and Discussion

### Plasmablasts promote Tfh cell expansion

**FIGURE 1.**
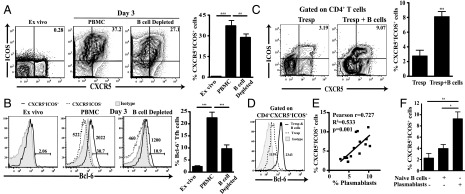
B cells promote human Bcl-6^+^ Tfh cells. PBMCs from healthy individuals (*n* = 8) were stained for Tfh cell markers CXCR5 and ICOS. B cell–depleted and whole PBMCs were stimulated with anti-CD3/CD28 for 3 d and stained for Tfh cell markers (*n* = 8). (**A**) Representative flow cytometry plots (gated on CD4) and cumulative data (mean ± SE) showing the frequency of Tfh cells. (**B**) Representative graphs and cumulative data (mean ± SE) showing Bcl-6 expression in Tfh cells (mean fluorescence intensity and % Bcl-6^+^ gated on CXCR5^+^ICOS^+^ T cells). (**C**) Purified Tresp cells from healthy individuals were stimulated or not with autologous B cells for 4 d with anti-CD3 and IL-4. Representative flow cytometry plots and cumulative data showing the percentage of Tfh cells (*n* = 12, mean ± SE). (**D**) Representative graphs of Bcl-6 expression in Tfh cells from cultures described in (C). (**E**) Correlation between the percentage of Tfh cells and plasmablasts from cultures described in (C). (**F**) Responder T cells from healthy individuals were cultured alone or with naive B cells or plasmablasts for 4 d, and the percentage of Tfh cells was determined (*n* = 5, mean ± SE). *p < 0.05, **p < 0.01, ***p < 0.001.

Human Tfh cells coexpress CXCR5 and ICOS at high density (4). In our study, Tfh cells were defined as CD4^+^CXCR5^+^ICOS^+^. Only a small fraction of circulating CD4^+^ T cells in healthy donors possessed these characteristics (Fig. 1A), as well as expressed PD1 but were negative for CD45RA (Supplemental Fig. 1A). Bcl-6 was expressed at low levels in circulating Tfh cells (Fig. 1B), consistent with previous findings (4). In contrast, when PBMCs were stimulated with anti-CD3/CD28, CXCR5 and ICOS expression greatly increased (Fig. 1A), as did Bcl-6 expression in Tfh cells (Fig. 1B). We sought to identify whether B cells promoted the formation of Tfh cells and found that removal of the former from PBMCs led to a significant reduction in Tfh cells (Fig. 1A), as well as Bcl-6 expression in the Tfh cell population, after stimulation with anti-CD3/CD28 (Fig. 1B), suggesting a role for B cells in the maintenance of this T cell lineage.

This finding prompted us to address whether B cells directly support the expansion of Tfh cells. Responder T (Tresp) cells (CD4^+^CD25^−^CD127^+^) were cultured with CD19^+^ B cells and stimulated with either anti-CD3 and IL-4 (Fig. 1C) or anti-CD3/CD28 (Supplemental Fig. 1B). The percentage of Tfh cells increased significantly in the presence of B cells under both conditions. These Tfh cells expressed high levels of Bcl-6 (Fig. 1D), as well as PD-1, CD200, and CD57 (data not shown). In these cocultures, the percentage of Tfh cells correlated with the percentage of plasmablasts (CD19^+^CD38^high^CD27^high^) (Fig. 1E) but not naive B cells (CD19^+^IgD^+^CD38^−/low^CD27^−^) or memory B cells (CD19^+^CD38^−^CD27^+^) (Supplemental Fig. 1C).

Given the close correlation between plasmablasts and Tfh cells, we hypothesized that this B cell subset was responsible for the increased frequency of Tfh cells. Therefore, plasmablasts were isolated from peripheral blood, according to their expression of CD38 and CD27 (5) (Supplemental Fig. 1D), and compared with naive B cells for their ability to expand Tfh cells. Plasmablasts, but not naive B cells, could drive the expansion of Tfh cells from Tresp cells (Fig. 1F). Our data reveal that not all circulating B cells are equally equipped to expand Tfh cells and identify the potent properties of plasmablasts in increasing Tfh cell numbers.

### Plasmablasts induce the differentiation of Tfh cells

**FIGURE 2.**
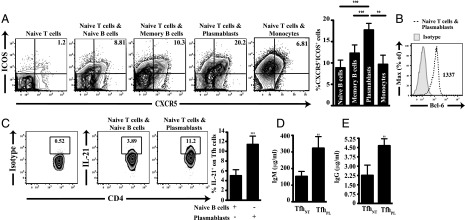
Plasmablasts induce the differentiation of Tfh cells. Naive T cells together with naive B cells, memory B cells, plasmablasts, or monocytes were stimulated with anti-CD3/CD28 for 4 d. (**A**) Representative flow cytometry plots (gated on CD4^+^ T cells) and cumulative data showing the percentage of Tfh cells (*n* = 35, mean ± SE). (**B**) Representative graphs showing Bcl-6 expression in Tfh cells induced in the presence of plasmablasts; mean fluorescence intensity value is indicated. (**C**) Representative flow cytometry plots and cumulative data showing the percentage of Tfh cells expressing IL-21 in cultures of naive T cells with naive B cells or plasmablasts (*n* = 9, mean ± SE). CD4^+^ T cells were repurified from cultures of naive T cells alone (Tfh_NT_) or with plasmablasts (Tfh_PL_) (day 4) and cocultured with freshly isolated autologous naive (**D**) or memory (**E**) B cells in the presence of Staphylococcal enterotoxin B for 10 d. IgM and IgG Ab production was measured by ELISA (*n* = 5, mean ± SE). **p < 0.001, ***p < 0.0001.

We next assessed whether circulating plasmablasts possessed the capacity to induce the differentiation of Tfh cells from naive T cells. Thus, plasmablasts, naive and memory B cells, and monocytes were isolated and added to naive T cells in the presence of anti-CD3/CD28. Plasmablasts were the most potent inducers of Tfh cell differentiation (Fig. 2A). Tfh cells induced in the presence of plasmablasts expressed high levels of PD-1 (Supplemental Fig. 1E) and Bcl-6 (Fig. 2B). In addition, plasmablast-induced Tfh cells secreted significantly more IL-21 compared with naive B cell–induced Tfh cells (Fig. 2C). Although naive B cells possessed some ability to induce Tfh cell differentiation, this could be explained, in part, by the formation of plasmablasts from naive B cells during the culture (Supplemental Fig. 1F). Indeed, the percentage of Tfh cells correlated with the numbers of plasmablasts present in these “naive B cell” cultures (Supplemental Fig. 1G). This finding suggests that the induction of Tfh cell differentiation was due, in part, to newly formed plasmablasts from naive B cells, although other mechanisms may contribute, given that monocytes can increase the number of Tfh cells above that seen with naive T cells alone. To support this finding we cocultured different ratios of naive CD4^+^ T cells/plasmablasts and assessed the induction of Tfh cell differentiation. The ratio of plasmablasts/naive CD4^+^ T cells in these cultures was directly proportional to Tfh cell differentiation (Supplemental Fig. 1H). To ascertain whether the differentiated Tfh cells were functional, T cells were repurified after 4 d of culture with plasmablasts and incubated with freshly isolated autologous naive B cells or memory B cells, as previously described (6). Plasmablast-induced Tfh cells provided enhanced B cell help and supported naive B cells and memory B cells to produce significantly more IgM (Fig. 2D) and IgG (Fig. 2E), respectively, compared with T cells that had not been exposed to plasmablasts. To our knowledge, our results show for the first time that human plasmablasts are potent inducers of Tfh cell differentiation. Of relevance, murine plasma cells were shown to inhibit, rather than promote, Tfh cell formation (7). This discrepancy may reflect differences between plasma cells and circulating plasmablasts, or it could be due to differential regulation of Tfh cells in mice and humans.

### Plasmablast-derived IL-6 induces Tfh cells

**FIGURE 3.**
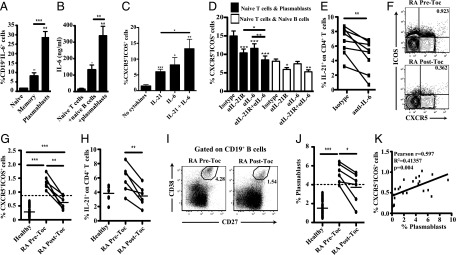
Plasmablast production of IL-6 induces Tfh cell differentiation. (**A**) Plasmablasts and naive and memory B cells from healthy PBMCs were stained for intracellular production of IL-6. Frequency of IL-6–secreting cells is shown (*n* = 9, mean ± SE). (**B**) IL-6 levels in supernatants collected 4 d after culture of naive T cells either alone or with naive B cells or plasmablasts stimulated with anti-CD3/CD28 (*n* = 9, mean ± SE). (**C**) Naive T cells from healthy individuals (*n* = 8) were stimulated with anti-CD3/CD28 for 6 d in the presence or absence of 10 ng/ml IL-21, IL-6, or a combination (cumulative data showing mean ± SE). (**D**) Addition of anti–IL-21R (αIL-21R) and/or anti–IL-6 (αIL-6) in cultures of naive T cells and plasmablasts or naive T cells and naive B cells (*n* = 11, mean ± SE). (**E**) In vitro cocultures of naive T cells and plasmablasts were treated with anti–IL-6–blocking Ab for 4 d, and CD4^+^ T cells were stained for IL-21 (*n* = 8). (**F**) Representative flow cytometry plots gated on CD4^+^ T cells showing the frequency of Tfh cells pre- and posttocilizumab therapy. (**G**) PBMCs from 41 healthy individuals and 6 paired patients with RA (pre- and posttocilizumab therapy) were stained ex vivo for Tfh cells. Cumulative data presented in a scatter dot plot showing mean percentage of Tfh cells in the CD4 compartment. (**H**) Cumulative data showing percentage of IL-21–producing CD4^+^ T cells. PBMCs from 32 healthy individuals and 6 paired RA patients (pre- and posttocilizumab therapy) were stained ex vivo for plasmablasts. (**I**) Representative flow cytometry plots showing the frequency of plasmablasts (percentages of CD27^high^CD38^high^ are indicated) pre- and posttocilizumab therapy. (**J**) Cumulative data showing mean percentage of plasmablasts. (**K**) The percentage of Tfh cells was correlated with the percentage of plasmablasts in RA patients receiving tocilizumab treatment (*n* = 22). *p < 0.05, **p < 0.01, ***p < 0.001.

In our efforts to elucidate the mechanism by which plasmablasts induce Tfh cell differentiation, we discovered that plasmablasts produce large amounts of IL-6, more than either naive or memory B cells (Fig. 3A, Supplemental Fig. 2A). Plasmablasts cultured with naive T cells yielded more IL-6 than did naive B cells and T cells or naive T cells alone (Fig. 3B). In addition, IL-6 detected in the coculture supernatants correlated with the percentage of Tfh cells (Supplemental Fig. 2B). Addition of IL-21 and IL-6 to naive T cells significantly induced Tfh cell differentiation (Fig. 3C, Supplemental Fig. 2C). Tfh cell differentiation was greater when a combination of IL-21 and IL-6 was used, confirming previous observations in mice whereby loss of both IL-6 and IL-21 led to a significant reduction in Tfh cell numbers (2, 3). Although IL-12 has been implicated in human Tfh cell differentiation (8), we could not detect IL-12 production by plasmablasts.

We next investigated whether the production of IL-6 and IL-21 mediated the differentiation of Tfh cells by plasmablasts. In vitro blockade of IL-21R or IL-6 in cocultures of naive T cells and plasmablasts significantly decreased the acquisition of a Tfh cell phenotype (Fig. 3D). Blockade of both cytokines further reduced the percentage of Tfh cells to the level found when naive B cells replaced plasmablasts in these cultures. Furthermore, IL-6 blockade in cultures of plasmablasts and naive T cells from healthy individuals also reduced IL-21 production by T cells (Fig. 3E), suggesting that IL-6 plays an important role in inducing Tfh cells through the stimulation of T cell IL-21 production.

### Tocilizumab therapy in patients with RA reduces Tfh cells and plasmablasts

To glean some insight into the in vivo relevance of our findings in patients, we investigated the interrelationships between Tfh cells and plasmablasts in patients with RA before and after tocilizumab therapy. Tocilizumab targets IL-6R and is an effective treatment for patients with RA (9). In agreement with previous studies (10), RA patients showed significantly more circulating Tfh cells compared with healthy individuals (Figs. 1A, 3F, 3G). Consistent with our in vitro data, RA patients responding to tocilizumab therapy showed a marked reduction in circulating Tfh cells (Fig. 3G). Paralleling our in vivo observations, CD4^+^ T cell IL-21 production was decreased significantly after treatment with tocilizumab (Fig. 3H). Tocilizumab treatment also reduced the plasmablast frequency in patients with RA (Fig. 3I, 3J). Moreover, there was a significant correlation between the percentage of Tfh cells and plasmablasts in patients with RA after tocilizumab treatment (Fig. 3K). These results suggest that tocilizumab could ameliorate disease by reducing Tfh cells and Ab-secreting cells in RA.

Our data implicate a previously unrecognized function of human circulating plasmablasts as potent inducers of Tfh cell differentiation via IL-6. One can envisage a feedback mechanism by which circulating plasmablasts traffic from one inflammatory site and create further foci of inflammation through the differentiation of Tfh cells, which, in turn, induces plasmablast formation. This feedback mechanism is tightly controlled under normal conditions; however, in infections and autoimmune diseases it could rapidly result in large numbers of Tfh cells and plasmablasts in several locations. This amplification loop represents an important therapeutic target in autoimmune diseases, such as RA, and it also could be boosted to improve responses to infection and to enhance vaccination strategies.

## Supplementary Material

Data Supplement
