# Supplementary material for: Cutting Edge: Circulating Plasmablasts Induce the Differentiation of Human T Follicular Helper Cells via IL-6 Production
Source: J Immunol. 2015 Feb 13;194(6):2482–5. doi: 10.4049/jimmunol.1401190 (PMC4356730; doi:10.4049/jimmunol.1401190)
Supplement: Data Supplement [file JI_1401190.zip › JI_1401190_Supplemental_Figures_1.pdf]

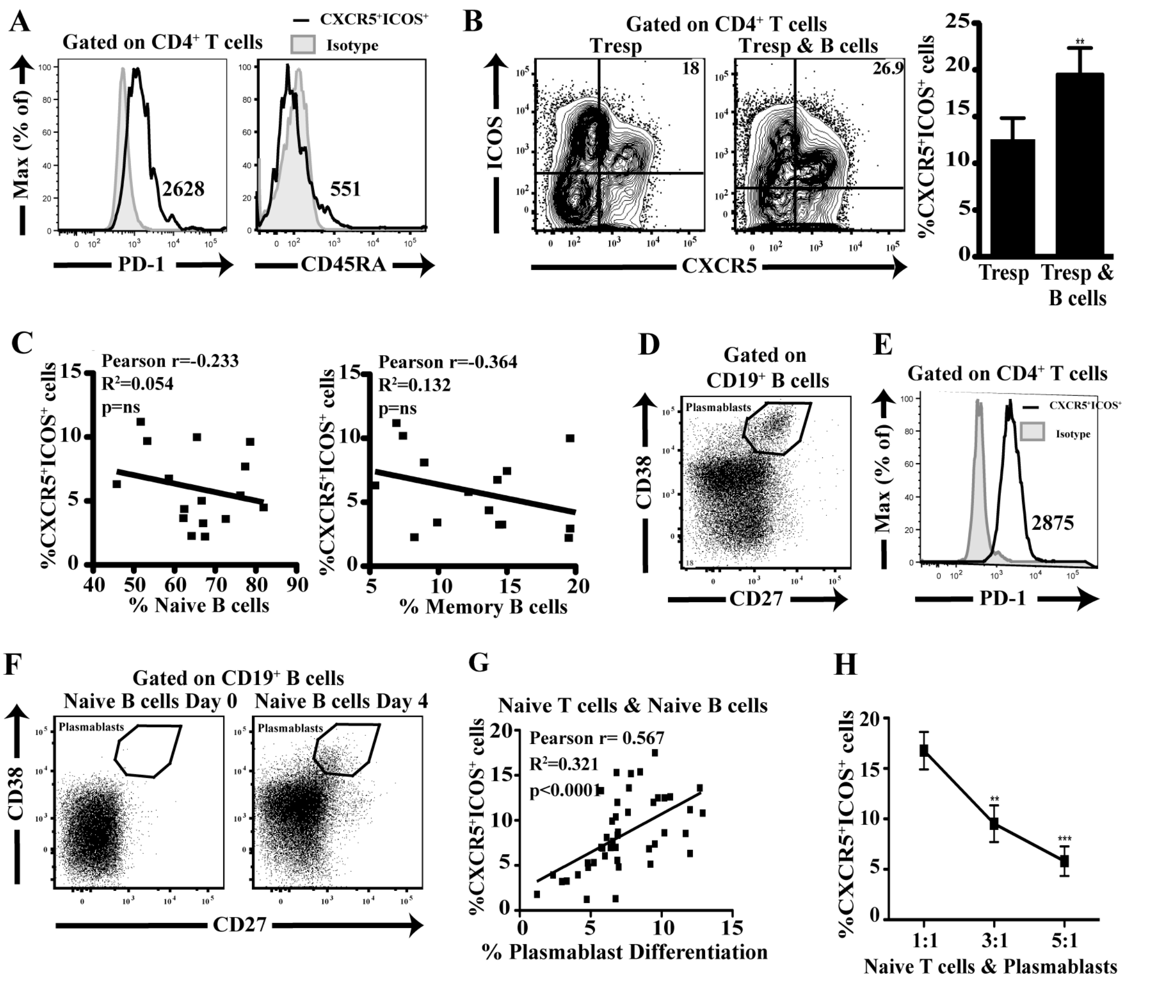

Supplementary Figure 1. (A) Representative histogram plots showing PD-1 and CD45RA expression on Tfh cells ex vivo, numbers indicate MFI. (B) Purified responder T cells from healthy individuals were stimulated ± autologous B cells for 4 days with anti-CD3/CD28. Representative flow cytometry plots and cumulative data showing the percentage of Tfh cells (n=6, mean±SE). (C) Correlation between the percentage of Tfh cells and naïve and memory B cells from cultures described in Figure 1C. (D) Representative flow cytometry plot indicating gating for plasmablasts based on CD27 and CD38 expression. (E) Representative histogram plots showing PD-1 expression on Tfh cells in vitro, numbers indicate MFI. (F) Representative flow cytometry plot indicating plasmablast differentiation in cultures of naïve B cells and naïve T cells stimulated with anti-CD3/CD28. (G) Correlation between Tfh cells and plasmablasts in cultures of naïve B cells and naïve T cells stimulated with anti-CD3/CD28 at day 4. (H) Plasmablasts were cultured together with different ratios of naive T cells from the same healthy individuals and the percentage of Tfh cells was measured. Cumulative data from healthy individuals (n=7) are shown (mean±SE). **$p<0.001$.

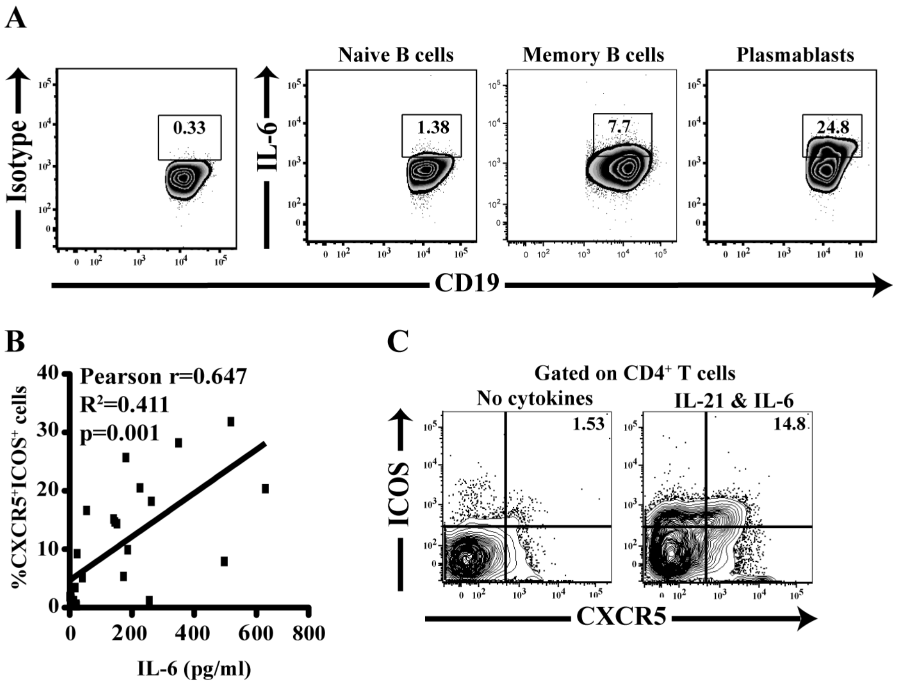

Supplementary Figure 2. (**A**) Plasmablasts, naïve and memory B cells from healthy PBMC were stained for intracellular production of IL-6 after 4 hour stimulation with PMA, ionomycin and Golgi Plug. Representative flow cytometry plots showing the frequency of IL-6 secreting B cells. **(B)** Correlation between IL-6 concentration and percentage of Tfh cells in cultures from Figure 3B. (**C**) Naïve T cells, from healthy individuals (n=8), were stimulated with anti-CD3/CD28 for 6 days in the presence or absence of 10ng/ml IL-21, IL-6 or a combination. Representative flow cytometry plots showing the frequency of Tfh cells with cytokines added as indicated. For cumulative data see Figure 3C in main text.
